# Microstructural and Thermal Transport Properties of Regioregular Poly(3-hexylthiophene-2,5-diyl) Thin Films

**DOI:** 10.3390/ma15217700

**Published:** 2022-11-02

**Authors:** Kai Herrmann, Simon Freund, Fabian Eller, Tamino Rößler, Georg Papastavrou, Eva M. Herzig, Markus Retsch

**Affiliations:** 1 Department of Chemistry, Physical Chemistry 1, University of Bayreuth, Universitätsstraße 30, 95447 Bayreuth, Germany; 2 Dynamics and Structure Formation—Herzig Group, Department of Physics, University of Bayreuth, Universitätsstraße 30, 95447 Bayreuth, Germany; 3 Physical Chemistry 2, University of Bayreuth, Universitätsstr 30, 95447 Bayreuth, Germany; 4 Bavarian Polymer Institute, Bayreuth Center for Colloids and Interfaces and Bavarian Center for Battery Technology (BayBatt), University of Bayreuth, Universitätsstraße 30, 95447 Bayreuth, Germany

**Keywords:** polymeric thin film, microstructure, morphology, thermal conductivity, poly(3-hexyl thiophene-2,5-diyl)

## Abstract

Polymeric thin films offer a wide range of exciting properties and applications, with several advantages compared to inorganic counterparts. The thermal conductivity of such thin films ranges typically between 0.1–1 W m${}^{-1}$ K${}^{-1}$. This low thermal conductivity can cause problems with heat dissipation in various applications. Detailed knowledge about thermal transport in polymeric thin films is desired to overcome these shortcomings, especially in light of the multitude of possible microstructures for semi-crystalline thin films. Therefore, poly(3-hexylthiophene-2,5-diyl) (P3HT) is chosen as a model system to analyze the microstructure and optoelectronic properties using X-ray scattering and absorption spectra along with the thermal transport properties using the photoacoustic technique. This combination of analysis methods allows for determining the optoelectronic and thermal transport properties on the same specimen, supplemented by structural information. The effect of different molecular weights and solvents during film preparation is systematically examined. A variation of the optoelectronic properties, mainly regarding molecular weight, is apparent, while no direct influence of the solvent during preparation is discernible. In contrast, the thermal conductivities of all films examined fall within a similar range. Therefore, the microstructural properties in the ordered regions do not significantly affect the resulting thermal properties in the sample space investigated in this work. We conclude that it is mainly the amorphous regions that determine the thermal transport properties, as these represent a bottleneck for thermal transport.

## 1. Introduction

Polymeric thin films have become increasingly important in recent decades and are now used in various applications. Their usage includes separation membranes for fuel cells, electrodes in batteries, or active layers in organic photovoltaics [1,2,3]. Low cost, mechanical flexibility, solvent processability, and tailor-made functionalities make polymeric thin films attractive alternatives compared to their inorganic counterparts. Conjugated polymers are particularly interesting as they exhibit a backbone chain of alternating single- and double bonds, leading to the formation of a delocalized π-electron system when adequately doped [4,5]. Delocalized π-electron systems often result in interesting optical and electronic properties [6]. A well-established class of conjugated polymers are polythiophenes. In polythiophenes, charge conduction occurs via intrachain and interchain charge transport in the crystalline regions, while the amorphous part conducts via hopping or tunneling [7,8,9]. Within the material class of polythiophenes, the most studied system is poly(3-hexylthiophene-2,5-diyl) (P3HT) due to its electrical and optical properties [7,10,11].

To extract information about the aggregation behavior of P3HT in thin films through a relatively simple measurement, Spano developed a model to describe the absorption from H-aggregates comprising parallelly aligned, cofacially packed conjugated chains in the case of weak excitonic coupling [12,13]. Using this model, the UV-vis absorption *A* as a function of the photon energy *E* results via [14]:

(1) $$A\left(E\right)\propto \sum_{m=0}\left(\frac{S^{m}}{m!}\right){\left(1-\frac{We^{-S}}{2E_{p}}\sum_{n\neq m}\frac{S^{n}}{n!n-m}\right)}^{2}\exp\left(-\frac{{\left(E-E_{0-0}-mE_{p}-0.5WS^{m}e^{-S}\right)}^{2}}{2\sigma^{2}}\right)$$

through transitions between the vibrational levels *m* and *n*. Herein, *S* is the Huang–Rhys factor, $E_{p}$—the intermolecular vibrational energy, $E_{0-0}$—the 0–0 transition energy, *W*—the exciton bandwidth, and σ—the Gaussian linewidth [15].

A characteristic feature of both bulk polymers and polymeric thin films is their low thermal conductivity, mainly in the order of 0.1–1 W m${}^{-1}$ K${}^{-1}$ [16]. Depending on the application, this low thermal conductivity can be disadvantageous if generated heat is not supposed to accumulate. It can also be advantageous if heat losses need to be prevented or a thermal gradient should be maintained. For P3HT, several investigations have examined the influence of film thickness, blending, or preparation parameters on their thermal transport properties [17,18,19,20]. As pointed out by many studies, a holistic understanding of thermal transport in polymer thin films is still missing. Up to now, most investigations have focused on the influence of structural properties such as the degree of crystallinity or the thin film thickness. Particularly in the case of semiconducting polymers, the interplay between the optoelectronic and thermal transport properties has not been investigated. Nevertheless, it is difficult to establish a consistent understanding since many parameters simultaneously influence the thermal properties of polymeric thin films. Our approach, consequently, is to relate the thermal properties to the structural properties.

This work, therefore, explores possible structure–property relationships between the thermal conductivity and nanostructure as well as morphology-related properties of P3HT thin films. We systematically investigate the optoelectronic and structural properties of three regioregular P3HT polymers with distinct molecular weights and solvent-processing conditions. After providing details on the employed materials and methods, the extracted morphologic information based on the absorption spectra and scattering analysis are presented. The set of samples analyzed using UV-vis absorption spectra is subsequently thermally analyzed using the photoacoustic technique, resulting in the direct determination of the thin film cross-plane thermal conductivity without requiring further measurements. We finally correlate our structural analysis to the thermal transport properties. Potential correlations are discussed, focusing on the fraction of aggregates and the exciton binding energy.

## 2. Materials and Methods

Regioregular P3HT with different molecular weights was purchased from Sigma-Aldrich (average $M_{w}$ 20–45 kg mol${}^{-1}$, 50–75 kg mol${}^{-1}$, and 85–100 kg mol${}^{-1}$ with regioregularity ≥ 90%) and used as received. Chlorobenzene, CB (anhydrous ≥ 99.8%), 1,2-dichlorobenzene, 1,2-DCB (anhydrous 99%) and 1,2,4-trichlorobenzene, 1,2,4-TCB (anhydrous ≥ 99%) were also purchased from Sigma-Aldrich and used as received.

Thin films were prepared by dissolving the respective polymer in the respective solvent at 100 °C for 20 min and spin-coating the solutions on Quartz substrates (Präzisions Glas und Optik GmbH). The spin-coating parameters are presented in Table 1. The films were annealed for about 16 h at 40 °C in a vacuum oven.

UV–vis absorption was measured with a UV–vis spectrometer (Cary 5000, Agilent Technologies) between 350 nm and 750 nm, corresponding to 1.65 eV to 3.54 eV, with an integration time of 0.1 ms in transmission geometry. To ensure a good resolution also at high absorption for thicker samples ($A_{\max}\geq 3$), the reference beam was attenuated using an attenuation grid and the integration time increased to 0.5 ms.

The data analysis on the absorption spectra was performed according to Ref. [14]. For the fit to Equation (1), a lower boundary of 1.95 eV and an upper boundary of 2.35 eV were applied using the Matlab function *nlinfit* employing the Levenberg–Marquardt algorithm [15]. The 95% confidence intervals were calculated using the Matlab function *nlparci* based on the residuals for the fitted model and the estimated variance-covariance matrix for the fitted coefficients.

Grazing incidence wide-angle X-ray scattering (GIWAXS) was performed on a laboratory system at the University of Bayreuth (Xeuss 3.0, Xenocs SAS, Grenoble, France) with a Cu Kα source (λ=1.54 Å), a Dectris EIGER 2R 1M detector, and a sample-to-detector distance of 72 mm. Scattering experiments were carried out at room temperature under vacuum on samples on Quartz substrates with a length of 4 mm. The incident angle was set to 0.20${}^{\circ }$ well above the critical angle of 0.16${}^{\circ }$, which probes the full depth of the films. The presented *q*-profiles are cake cuts covering an azimuthal angle of 70–110${}^{\circ }$ for the cuts in the vertical direction and 0–20${}^{\circ }$ as well as 160–180${}^{\circ }$ for the cuts in the horizontal direction.

The data analysis is based on fitting the horizontal and vertical cuts. All performed fits are Pseudo-Voigt fits, described by the following expression for a single peak:

(2) $$f\left(q\right)=A\cdot \left[\eta \cdot L(q)+(1-\eta )\cdot G(q)\right],\;\mathrm{with}\;0<\eta <1$$

(3) $$G\left(q\right)=\exp\left[-\ln\left(2\right)\cdot {\left(\frac{q-c}{b}\right)}^{2}\right],\;L\left(q\right)=\frac{1}{1+{\left(\frac{q-c}{b}\right)}^{2}}$$

where *A* is the peak amplitude, *c* is the peak position, 2b is the full width at half maximum of the Pseudo-Voigt peak, and η the Pseudo-Voigt mixing parameter. To fit the superposition of various peaks, we fitted the sum of five Pseudo-Voigt peaks for both directions. Moreover, background scattering was fitted with the functional form of $d_{h}\cdot q^{-4}+e_{h}$ in the horizontal and of $d_{v}\cdot q^{-5}+f\cdot q^{-2}+e_{v}$ in the vertical direction, where $d_{h}$, $e_{h}$, $d_{v}$, $e_{v}$, and *f* are constants. For the fitting, we used *lmfit* in Python. Normalization of the peak amplitude is based on the absorption spectra of the respective samples. The areas of the aggregate and amorphous fit are added, while the aggregate area is divided by 1.39 due to its higher molar extinction coefficient [21].

The samples for thermal transport characterization and a thermally thick reference material (quartz) were coated with a 100 nm gold layer by thermal evaporation to ensure high and near-surface absorption for the photoacoustic characterization. The layer thickness was monitored using a quartz crystal microbalance. Photoacoustic measurements were performed with a continuous wave Coherent Genesis MX488-1000, Utrecht, The Netherlands laser. The laser was modulated with a ConOptics 350-160 electro-optic modulator, operated by a sinusoidal signal of a Zurich Instruments lock-in amplifier HF2LI, Zurich, Switzerland. The acoustic signal was measured using a Bruel & Kjaer 4398-A-011, Bremen, Germany microphone, which is subsequently demodulated in the lock-in amplifier.

The pressure in the photoacoustic cell was set to 1.379 bar of helium, corresponding to 20 psi. A comprehensive explanation of the experimental setup is given in Ref. [22] for more practical information.

The data analysis on the photoacoustic measurements is performed according to Ref. [22]. To determine the sample’s thermal properties using the multilayer model from Hu et al., the thermal properties of the substrate (quartz) and transducer (gold) are required [23]. Therefore, the thermal effusivity and diffusivity of gold are taken as $\varepsilon_{\mathrm{Au}}$ = 22,271 W s${}^{1/2}$ m${}^{-2}$ K${}^{-1}$ and $D_{\mathrm{Au}}=8.06\times 10^{-5}$ m${}^{2}$ s${}^{-1}$ [24]. The thermal effusivity and diffusivity of quartz are taken as $\varepsilon_{\mathrm{Quartz}}=1499.8$ W s${}^{1/2}$ m${}^{-2}$ K${}^{-1}$ and $D_{\mathrm{Quartz}}=8.47\times 10^{-7}$ m${}^{2}$ s${}^{-1}$ [24]. Again, the least-squares fitting function *nlinfit* is used to determine the sample’s thermal effusivity and diffusivity. The thermal conductivity is subsequently calculated from these parameters. The approach for error estimation is described in Ref. [22]. In doing so, two independent measurements are analyzed using a Monte Carlo approach for the controlled parameters sample and transducer thickness. Therefore, 1000 iterations with randomly selected controlled parameters on two data sets, measured on the same sample but at different positions, were performed. Simultaneously, the uncertainty of every fit procedure is taken into account by the respective residuals and the estimated variance–covariance matrix.

The thicknesses of the polymeric films were determined using an Olympus OLS5000, Hamburg, Germany laser confocal microscope and a 50× microscope objective. The error is calculated by measuring five areas of 260 μm × 260 μm at the top, left, center, right, and bottom of the circular area with a radius of 2 mm probed by the photoacoustic measurement and assuming a Gaussian distribution.

## 3. Results

To enable both the optical and the photoacoustic characterization, specific requirements for the sample thickness have to be met. For optical characterization, the samples should not be excessively thick to ensure a detectable transmission. In contrast, due to the limited frequency regime of the photoacoustic technique, the samples should have a certain thickness to allow the significant determination of the thermal conductivity [22]. Therefore, the film thickness should be about 500 nm to allow quantitative measurements of both optoelectronic and thermal properties. This is also an adequate thickness for GIWAXS characterization [25,26]. Quartz substrates are well suited for all these measurements because they exhibit high optical transparency in the wavelength regime of interest and thermal effusivity in a similar order of magnitude as polymers.

**Figure 1:**
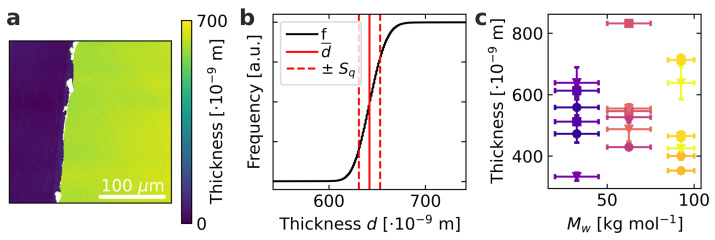
(**a**) Exemplary confocal microscopy image of ${\bar{M}}_{w}$ = 32.5 kg mol${}^{-1}$ spin-coated from chlorobenzene. The height is color-coded, visualizing the quartz substrate as blue and the thin film as green. (**b**) Exemplary cumulative frequency distribution *f* as a function of thickness, as well as the average thickness $\bar{d}$ and the surface roughness $S_{q}$ calculated from subfigure (**a**). (**c**) Resulting film thicknesses, errors are derived from the inhomogeneities at different measurement points. Circular symbols represent films spin-coated from chlorobenzene, squares films from 1,2-dichlorobenzene, and triangles films from 1,2,4-trichlorobenzene.

First, the parameters displayed in Table 1 were determined to produce films in the desired thickness range from the different solvents chlorobenzene, 1,2-dichlorobenzene, and 1,2,4-trichlorobenzene to achieve a variation in the microstructure [21,27]. An exemplary resulting film for the molecular weight of 32.5 kg mol${}^{-1}$ spin-coated from chlorobenzene is shown in Figure 1a. The polymeric films are partially removed from the substrate by scraping off with a glass pipette to produce a sharp edge. The color code here contains information about the z-axis, where the height of the substrate is normalized to zero. Based on this, the film thickness can be determined as shown with a cumulative frequency distribution in Figure 1b. In addition to the average film thickness, the surface roughness can also be determined from the respective measurements as a further quality characteristic. For this, we use the root mean square roughness $S_{q}$ as one of the most widely used ones as defined by

(4) $$S_{q}=\sqrt{\frac{1}{A}\underset{A}{\;\int \int \;}Z^{2}\left(x,y\right)\partial x\partial y},$$

where *A* represents the evaluated area and *Z* the respective height [28]. The parameter $S_{q}$, therefore, corresponds to the standard deviation of the height distribution. For all samples examined, $S_{q}$ is in the range of 10 nm to 40 nm, allowing a proper data analysis. This surface roughness is corroborated by atomic force microscopy measurements on P3HT samples spin cast from chlorobenzene solutions. The height images reveal a granular surface topology with undulations of a few 10 nm (Figure S1). Neither in the height, nor in the phase image were we able to resolve microcrystalline regions regardless of the molecular weight.

For the cross-plane thermal conductivity determination, however, not only the local surface roughness is of interest, but especially inhomogeneities and fluctuations of the film thickness in the investigated measuring area, which corresponds to approximately 12.6 mm${}^{2}$ [22]. To represent these inhomogeneities realistically, the film thickness was determined at five locations in the area of the thermal measurement, and the mean value and standard deviation were determined under the assumption of a Gaussian distribution. The film thickness results obtained from this procedure are shown in Figure 1c. To map a sample to sample variation, two separate films were examined for every combination of molecular weight and solvent. All produced films are in the required range between 330 nm to 830 nm, ensuring a significant analysis of optoelectronic and thermal properties.

**Figure 2:**
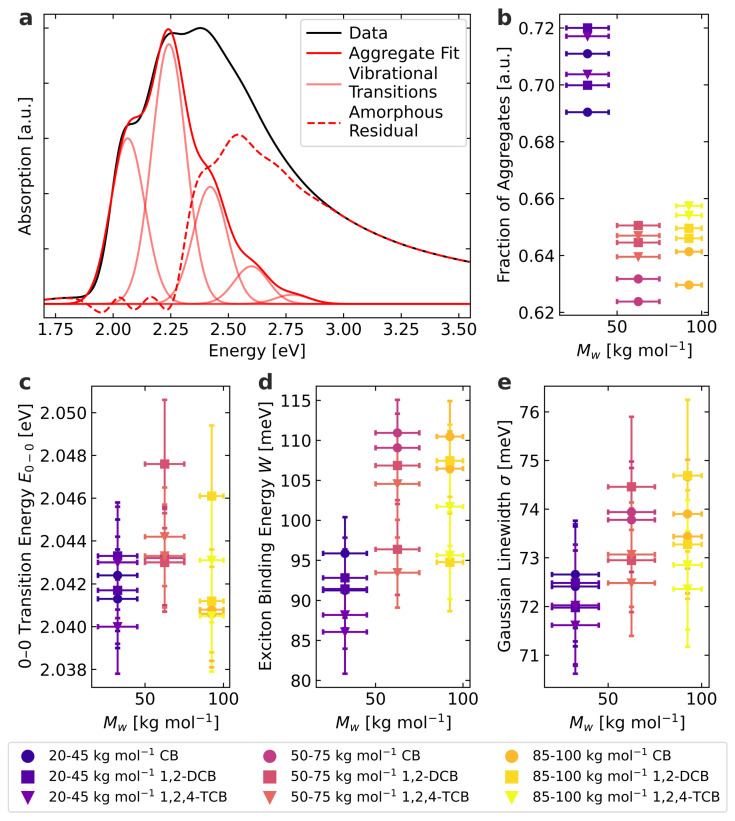
Performed UV-vis absorption spectra data analysis: (**a**) Exemplary fit on the absorption spectrum of ${\bar{M}}_{w}$ = 32.5 kg mol${}^{-1}$ spin-coated from chlorobenzene. The individual vibrational transitions sum up to the aggregate fit. (**b**–**e**) Extracted fraction of aggregates, 0–0 transition energy, exciton binding energy and Gaussian linewidth.

Having verified the fundamental requirements for the thin films, we can now turn to optoelectronic characterization. The acquired absorption spectra are evaluated according to Equation (1), which is demonstrated for the example of a molecular weight of 32.5 kg mol${}^{-1}$ spin-coated from chlorobenzene in Figure 2a. For the data analysis, *W*, $E_{0-0}$, σ, and a global proportionality factor were varied as free parameters, while *S* was taken as 1.0 and $E_{p}$ was taken as 0.179 eV, as reported in the literature [21,29,30]. Certain deviations between the model and measured values can be seen, but they are taken into account by the uncertainty estimation discussed in Section 2. The most common deviations are based on the model’s assumptions, simplifying the actual situation. It, therefore, would be expected that the amorphous residual is unstructured. However, it can be seen that the amorphous contribution resulting from the analysis exhibits structuring [15]. This structuring above approximately 2.5 eV is most likely an artifact due to electronic transitions at higher energies [12,14,15]. Furthermore, slightly structured residuals in the energy range below 2.5 eV are recognizable, suggesting that the Gaussian disorder is too simple to describe the thin films’ absorption spectrum fully [14,15]. Despite these deviations, the model used is nevertheless a practical possibility to estimate certain microstructural properties of the thin films.

The fraction of aggregates extracted from the absorption spectra is displayed in Figure 2b and calculated from the numerically integrated absorptions of the aggregate fit and the amorphous residual. Normalizing the aggregate integral with the factor of 1.39 is based on the different extinction coefficients of aggregated and non-aggregated P3HT [31,32]. The low molecular weight (${\bar{M}}_{w}$ = 32.5 kg mol${}^{-1}$) exhibits the highest fraction of aggregates, with the two higher molecular weights (${\bar{M}}_{w}$ = 62.5 kg mol${}^{-1}$ and ${\bar{M}}_{w}$ = 92.5 kg mol${}^{-1}$) being at a lower and similar level. Furthermore, it can be seen that the different films of the same molecular weight polymer display slightly different microstructures. However, we could not establish a direct and unambiguous correlation between the solvent used for spin-coating and the fraction of aggregates.

There is an inverted dependency for the extracted fit parameters in Figure 2c–e. Generally, the low molecular weight exhibits the lowest 0–0 transition energy, exciton binding energy, and Gaussian linewidth, while the two higher molecular weights are at a higher and similar level. Again, no direct influence of the solvent is discernible. The 0–0 transition energy is related to the peak position of the first vibrational transition in Figure 2a. The similar transition energies for the two higher molecular weights suggest that the local electronic properties of the chains in the ordered domains are only weakly affected by the molecular weight in this range [14]. The lower 0–0 transition energy is possibly due to fewer local torsions of the conjugated backbones within the aggregates for the low molecular weight [14]. Still, the relative differences appear to be minor. The exciton binding energy is related to the relative intensities of the vibrational transitions. It can be interpreted as a measure of the average conjugation length of planarized chain segments in the ordered domains [33]. A lower exciton binding energy is related to a higher conjugation length above a certain minimum length, which is the case for all films examined here [33]. Therefore, the fraction of aggregates is higher for the low molecular weight, and the conjugation length inside the aggregate domains is increased. The Gaussian linewidth is a measure of the energetic disorder inside the ordered domains and is related to the width of the vibrational transitions in Figure 2a. Again, the energetic disorder is the lowest for the lower molecular weight, while for the two higher molecular weights, it is higher and on a similar level.

In summary, the lower molecular weight exhibits the highest fraction of aggregates, the highest average conjugation length, and the lowest energetic disorder. The two higher molecular weights are more disordered in all respects, with both being at a similar level. The apparent cause is probably the increased entanglements in the forming film and the number of refolded or bridging polymer chains between the aggregates [14,34,35]. In any case, the used spin-coating solvent plays a minor role in influencing the optoelectronic properties compared to the molecular weight for our preparation parameters.

While the UV-vis absorption measurements examine the electronic interaction of neighboring polymer chains, we can characterize the stacking of polymer chains using x-ray diffraction. To examine the influence of molecular weight and processing solvent on the nanostructure beyond individual chains, we perform GIWAXS measurements.

**Figure 3:**
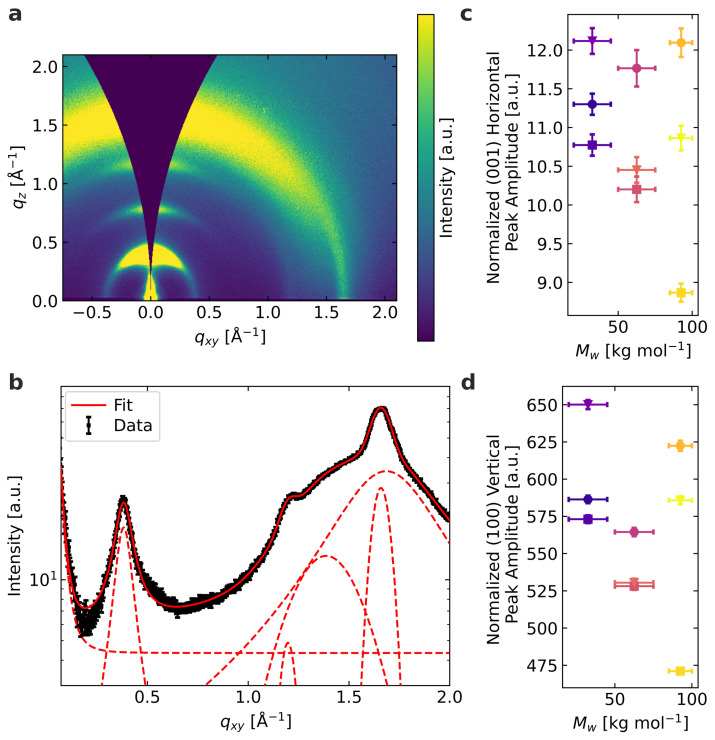
Performed GIWAXS analysis: (**a**) 2D GIWAXS data of ${\bar{M}}_{w}$ = 92.5 kg mol${}^{-1}$ spin-coated from 1,2,4-trichlorobenzene. (**b**) Horizontal cut of the 2D data showing the individual fitting contributions. (**c**) Normalized peak amplitude of the horizontal π-π stacking signal (001). (**d**) Normalized peak amplitude of the vertical lamellar stacking signal (100). The shown data sets are carried out on samples with a comparable thickness. The obtained peak amplitudes are normalized with the amount of examined material from individual absorption measurements.

In Figure 3a, example 2D GIWAXS data are displayed. P3HT is known to stack in two directions within a single crystallite, i.e., along the side chains (lamellar stacking) and by stacking the backbones via π-π stacking. As the short intermolecular distance of the π-π stacking enables electronic coupling, it more strongly regulates the optoelectronic and thermal properties than the lamellar stacking with its larger intermolecular distance across the non-conductive hexyl sidechains. Therefore, the lamellar stacking serves as an indicator for the aggregate quantity and quality, but the π-π stacking is expected to relate to transport properties.

We examine both stacking directions using GIWAXS. The spin-coating procedure yielded P3HT films with a variation in layer thicknesses, as outlined in Figure 1c. Therefore, we used the weighted combination of amorphous (weight = 1.39) and aggregated (weight = 1.0) material obtained from the individual absorption spectra of each film as a measure for the amount of scattering polymer in the X-ray beam to normalize the peak amplitudes. We are then able to use the normalized peak amplitudes for a quantitative comparison of stacking features between different molecular weight samples. For all samples, in the vertical direction at about q=0.39 Å${}^{-1}$ ((100) peak, d=16 Å), 0.78 Å${}^{-1}$ ((200) peak), and 1.16 Å${}^{-1}$ ((300) peak), the first three orders of the lamellar stacking scattering are clearly visible. In the horizontal direction, only the (100) lamellar peak stands out significantly from the background. Moreover, at about q=1.66 Å${}^{-1}$ (d=3.79 Å) in the horizontal direction, the well-defined π-π peak can be observed. This is a typical signature of edge-on-oriented P3HT. Between about 1.2 Å${}^{-1}$ and 1.7 Å${}^{-1}$, scattering of disordered P3HT can be seen, but also scattering of the Quartz substrate (reference measurement, see Figure S4) is contributing to the same *q*-range, especially in the vertical direction. To extract information on the lamellar and π-π stacking, we include the broad peaks of the disordered P3HT and Quartz underneath the π-π stacking peak into our fitting routine. The fit of the horizontal cut of the example data from Figure 3a in the horizontal direction is displayed in Figure 3b.

In Figure 3c,d, the normalized peak amplitudes of the (001) peak in the horizontal direction (π-π stacking) and the (100) peak in the vertical direction (dominant lamellar peak) are shown. The normalized peak amplitudes resemble the amount of material involved in π-π stacking and lamellar stacking, respectively. In all samples, we observed material containing polymer π-π stacking and lamellar stacking. However, we could not determine a systematic trend concerning the relative amounts among the different samples. Neither the different molecular weights, nor the various spin-coating solvents resulted in a systematic trend favoring one or the other stacking type quantitatively. The variability of the normalized peak amplitudes is highest for samples with the highest molecular weight, indicating that this polymer seems the most challenging to reproduce the nanostructures in a controllable way.

In contrast to the optical analysis presented in Figure 2, the GIWAXS data do not confirm a higher degree of order, in particular, in the case of the low molecular weight species. This does not contradict the optoelectronic properties, but is based on the differences in length scales probed. In Figures S5a and S4b, the peak widths of the same peaks are displayed. For the π-π stacking as well as the lamellar stacking, the differences between the values for the various solvents and molecular weights are rather small, and no systematic behavior is observable. The peak width is a measure for the range of ordering, where a smaller peak width signals a longer-range order. Neither for the lamellar, nor the π-π stacking does the peak width indicate the highest order for the low molecular weight as observed in the optoelectronic characterization, where shorter length scales are probed. Therefore, we conclude that despite systematic differences in the backbone ordering and energetic disorder, this does not translate to a systematic impact on the longer-range π-π and lamellar stacking, as measured by GIWAXS.

**Figure 4:**
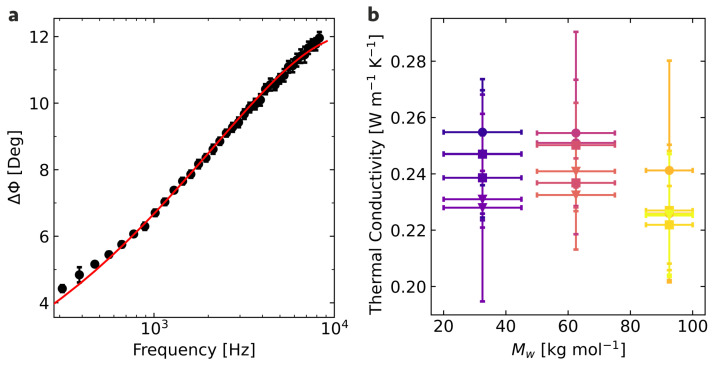
Performed photoacoustic data analysis: (**a**) Exemplary fit on the measured phase shift of ${\bar{M}}_{w}$ = 32.5 kg mol${}^{-1}$ spin-coated from chlorobenzene. (**b**) Extracted thermal conductivities for the different molecular weights and solvents. Circular symbols represent films spin-coated from chlorobenzene, squares films from 1,2-dichlorobenzene, and triangles films from 1,2,4-trichlorobenzene.

Having discussed the microstructural properties, we now turn to the thermal properties. An exemplary measurement of the photoacoustic phase shift Δϕ as a function of frequency, and the performed multilayer fit, are shown for the molecular weight of 32.5 kg mol${}^{-1}$ spin-coated from chlorobenzene in Figure 4a. The frequency position in combination with the sample thickness determines mainly the thermal diffusivity, while the phase shift values determine mainly the thermal effusivity in the one-dimensional limit of the thermal diffusion equation, which can be applied here [22,36]. The combination of both parameters then provides the thermal conductivity. The thermal conductivity for all samples investigated in this work is reported in Figure 4b.

The primary sources of errors are the inhomogeneities and fluctuations in the film thickness. Since layer thickness is one of the most critical parameters of the multilayer model, these uncertainties directly affect the resulting thermal conductivity. The thermal conductivity for all samples is between 0.22 W m${}^{-1}$ K${}^{-1}$ and 0.26 W m${}^{-1}$ K${}^{-1}$, which are generally in line with or slightly above literature values [18,20]. Furthermore, no thickness dependence effects are apparent for the investigated thickness regime, as shown in the Supporting Information (Figure S2). No direct influence of the solvent on the resulting properties is discernible for the thermal properties, similar to the microstructural properties. However, the impact of the molecular weight seems to be different here. The two lower molecular weights appear to be on a similar level at approximately 0.24 W m${}^{-1}$ K${}^{-1}$ on average, while the high molecular weight exhibits an average thermal conductivity of roughly 0.23 W m${}^{-1}$ K${}^{-1}$. This molecular weight dependency contrasts with the microstructure parameters, where the two higher molecular weights are at a similar level, while the lowest molecular weight deviates from them. Nevertheless, the relative deviations in thermal conductivity between the different molecular weights are comparatively small.

Since all structural, optoelectronic, and thermal transport properties have been determined on the same specimen, we can now correlate these properties to identify possible relationships.

## 4. Discussion

**Figure 5:**
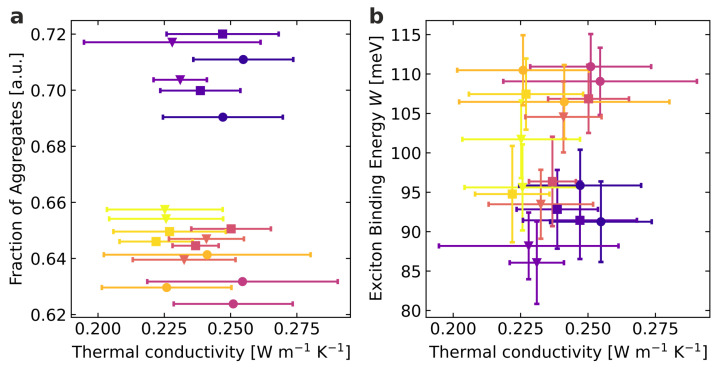
Correlations between extracted microstructural properties and thermal conductivity: (**a**) Relation between the fraction of aggregates and the thermal conductivity. (**b**) Relation between the exciton binding energy and the thermal conductivity. Color code: violet: low ${\bar{M}}_{w}$ samples, red: medium ${\bar{M}}_{w}$ samples, yellow: high ${\bar{M}}_{w}$ samples. Circular symbols represent films spin-coated from chlorobenzene, squares films from 1,2-dichlorobenzene, and triangles films from 1,2,4-trichlorobenzene.

Correlations between the microstructural properties, determined by absorption spectra analysis, and thermal properties determined on the same samples are investigated below. The microstructural properties are plotted against the thermal conductivity to identify possible correlations or independencies. Figure 5 shows this for the fraction of aggregates and the exciton binding energy.

Within the accuracy of the measurement, we do not find a correlation between the fraction of aggregates and the thermal conductivity in the investigated range of molecular weights and generated microstructures. Although a significant variation in the fraction of aggregates can be seen depending on the molecular weight, it does not affect the thermal conductivity. The thermal conductivity proves robust concerning the present fraction of aggregates and is, therefore, not affected in the evaluated domain. This is supported by the structural investigations on the longer length scales obtained from the GIWAXS measurements. While the fraction of aggregates obtained from spectroscopy correlates with the trend of peak amplitudes with molecular weight and solvent, the peak widths of the scattering data, which correlate with the length scale of ordered domains, do not show any systematic changes. For the exciton binding energy, a more complex situation emerges. For the high molecular weight, a similar situation as for the fraction of aggregates is present, where similar thermal conductivities for different exciton binding energies can be identified. In contrast, for the low and especially medium molecular weight, one could infer a connection between the exciton binding energy and the thermal conductivity. Slightly higher thermal conductivities accompany higher exciton binding energies. Considering the scatter and accuracy of our data, such a potential trend needs to be interpreted cautiously. Taking into account the 0–0 transition energy and the Gaussian linewidth, this trend is not confirmed (Supporting Information, Figure S3). Overall, we have to conclude that a correlation between the structure, the optoelectronic properties, and the thermal conductivity cannot be resolved in the P3HT thin films investigated here.

We interpret the main obstruction to a consistent identification of correlations between the microstructural properties and the thermal conductivity by the lack of sufficiently high difference in the thermal conductivity of the crystalline and amorphous regions, respectively. The fundamental factor underlying these results is the semi-crystalline structure of the investigated thin films. The amorphous regions mainly affect macroscopic thermal transport in semi-crystalline polymeric thin films with molecular weights above the critical entanglement molecular weight [37,38,39]. The critical entanglement molecular weight for P3HT is assumed to be approximately 35 kg mol${}^{-1}$ [37,40,41]. Especially for the high concentrations used in this work during spin-coating to enable a sufficient film thickness for the thermal characterization, chain entanglements are likely to have a very significant influence. The molecular weights used in this work are either on the order or above the critical entanglement molecular weight, which presumably leads to the amorphous domains forming the bottleneck for macroscopic thermal transport. Even though preferential chain orientation can significantly enhance the thermal conductivity of the amorphous phase, mostly through increased short-range ordering while long-range order remains absent, this is only achieved with special preparation procedures [42]. The amorphous phase for the relatively high film thickness and preparation by spin-coating examined in this work is, therefore, not considered to be oriented.

In contrast, the extracted microstructural properties are assigned to the ordered regions, as only these are included in the optoelectronic model and detectable in X-ray diffraction [12]. This reinforces our findings that a change in the microstructural properties in the ordered regions does not affect the resulting macroscopic thermal transport properties in the examined range of molecular weights. The bottleneck for macroscopic thermal transport remains the amorphous regions, which separate the ordered parts.

## 5. Conclusions

This work investigated possible relationships between nanostructure, optoelectronic, and thermal transport properties for regioregular P3HT thin films. Three different molecular weights (${\bar{M}}_{w}$ = 32.5 kg mol${}^{-1}$, 62.5 kg mol${}^{-1}$, and 92.5 kg mol${}^{-1}$) and three different solvents (chlorobenzene, 1,2-dichlorobenzene, and 1,2,4-tricholorobenzene) were chosen for this purpose. Absorption spectra were analyzed using the model from Spano, which showed that the two higher molecular weights exhibit similar optoelectronic properties [12]. In contrast, the lower molecular weight shows slight differences with a higher fraction of aggregates and a lower 0–0 transition energy, a lower exciton binding energy, and a lower Gaussian linewidth, corresponding to the highest degree of order in the crystalline domains. The spin-coating solvent used played a minor role in influencing the optoelectronic properties compared to the molecular weight of the P3HT. GIWAXS measurements also support this behavior. The spatial extension of the ordered domains shows no systematic changes with molecular weight or processing solvent. The thermal conductivity was determined on the same set of samples, revealing very similar values in the range of 0.22–0.26 W m${}^{-1}$ K${}^{-1}$. The fraction of aggregates proved to be unrelated to the thermal conductivity in the investigated sample space. In general, we find the microstructural properties not to be related to the thermal conductivity. This can be explained by the fact that non-oriented amorphous regions separate the ordered regions. These apparently dictate the macroscopic heat transport. The optoelectronic properties, however, are mainly governed by the ordered parts. Consequently, we cannot resolve an unambiguous relation between the optoelectronic and the thermal transport properties. Therefore, we would like to further encourage the field to explore possible relationships between microstructure and thermal properties, at best with a stronger focus on the amorphous region. This will be of immediate relevance for a range of polymer-based materials, where the thermal performance strongly depends on the structural arrangement across several length scales. Important related materials are polymer-based photovoltaics, organic light-emitting diodes, hybrid thermoelectric systems, or thermal interface materials. Similar to the structure of P3HT investigated in this study, these materials comprise ordered and disordered regions with significant contributions of the involved interfaces. Efficient heat transfer between the various compartments of these materials will only be possible with stringent control of the polymer morphology in each of these nanostructured spaces and requires a better fundamental understanding of this intricate interplay.

## Figures and Tables

**Table 1:** Utilized spin-coating parameters (concentration *c* and rotation speed ω) for all combinations of molecular weights and solvents.

Solvent	${\bar{M}}_{w}$ = 32.5 kg mol${}^{-1}$	${\bar{M}}_{w}$ = 62.5 kg mol${}^{-1}$	${\bar{M}}_{w}$ = 92.5 kg mol${}^{-1}$
Chlorobenzene	c=50 mg mL${}^{-1}$, ω=750 rpm	c=50 mg mL${}^{-1}$, ω=750 rpm	c=30 mg mL${}^{-1}$, ω=500 rpm
1,2-Dichlorobenzene	c=55 mg mL${}^{-1}$, ω=500 rpm	c=55 mg mL${}^{-1}$, ω=500 rpm	c=55 mg mL${}^{-1}$, ω=1000 rpm
1,2,4-Trichlorobenzene	c=70 mg mL${}^{-1}$, ω=500 rpm	c=70 mg mL${}^{-1}$, ω=500 rpm	c=70 mg mL${}^{-1}$, ω=1000 rpm

## Data Availability

The datasets generated and analyzed during the current study are available from the corresponding author on reasonable request.

## Supplementary Materials

The following are available online at https://www.mdpi.com/article/10.3390/ma15217700/s1, Figure S1: Height and phase AFM images of P3HT films, Figure S2: Thickness Dependency of Thermal Conductivity, Figure S3: Additional Correlations of Microstructure and Thermal Conductivity. Figure S4: GIWAXS of Quartz Reference. Figure S5: Additional Extracted GIWAXS Parameters.
