# Supplementary material for: Microstructural and Thermal Transport Properties of Regioregular Poly(3-hexylthiophene-2,5-diyl) Thin Films
Source: Materials (Basel). 2022 Nov 2;15(21):7700. doi: 10.3390/ma15217700 (PMC9654977; doi:10.3390/ma15217700)
Supplement: Supplementary file 1 [file materials-15-07700-s001.zip › materials-1977741-supplementary.pdf]

Supplementary Materials: Microstructural and Thermal Transport Properties of Regioregular Poly(3-hexylthiophene-2,5-diyl) Thin Films

Kai Herrmann [1], Simon Freund [1], Fabian Eller [2], Tamino Rößler [3], Georg Papastavrou [3,4], Eva M. Herzig [2] and Markus Retsch [1,4,*]

S1. Height and phase atomic force microscopy images

Height and phase images of spin cast P3HT films from chlorobenzene are shown in Figure S1. The granular surface exhibits height undulations of a few 10 nm. P3HT lamellar stacks could not be resolved.

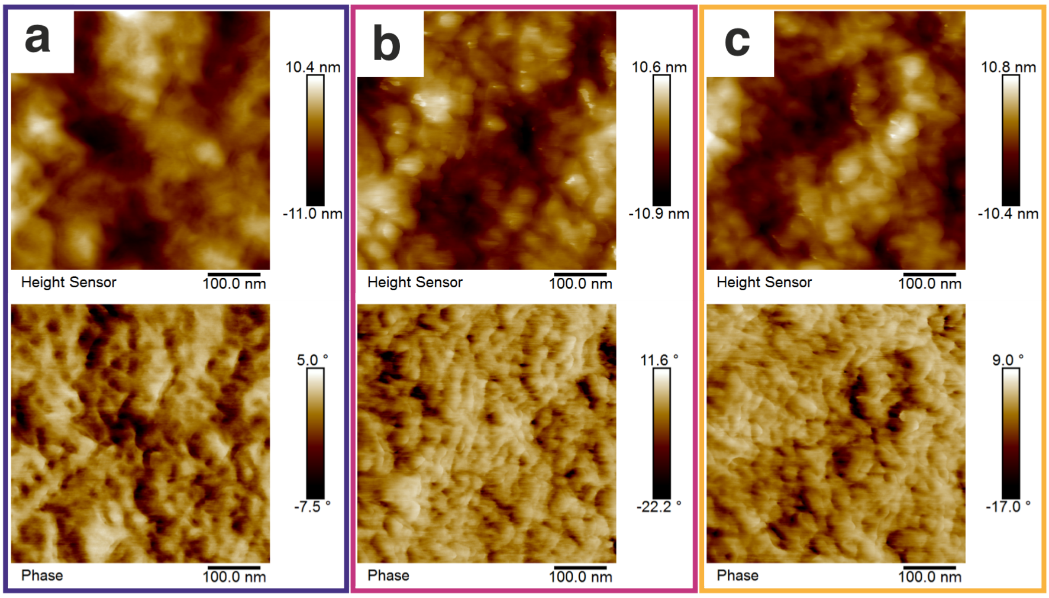

Figure S1. AFM measurement of the surface topography of spin cast films. a) $\overline{M}_w$=32.5 kg mol^{-1}, b) $\overline{M}_w$=62.5 kg mol^{-1}, c) $\overline{M}_w$=92.5 kg mol^{-1}. All AFM images were acquired at a Bruker Dimension Icon equipped with a Nanoscope V Controller. Tapping mode was applied under ambient conditions (f_{nom} = 300 kHz, k_{nom} = 26 N m^{-1}). Topography and phase data is depicted.

S2. Thickness Dependency of Thermal Conductivity

The thickness dependence of the thermal conductivity for all samples examined in this work is shown in Figure S2. No influence of the film thickness can be seen here, which verifies that the thermal conductivity in the investigated thickness range is independent of the film thickness. Typically, the influence of the film thickness on the thermal conductivity of polymers is only present below 100 nm.

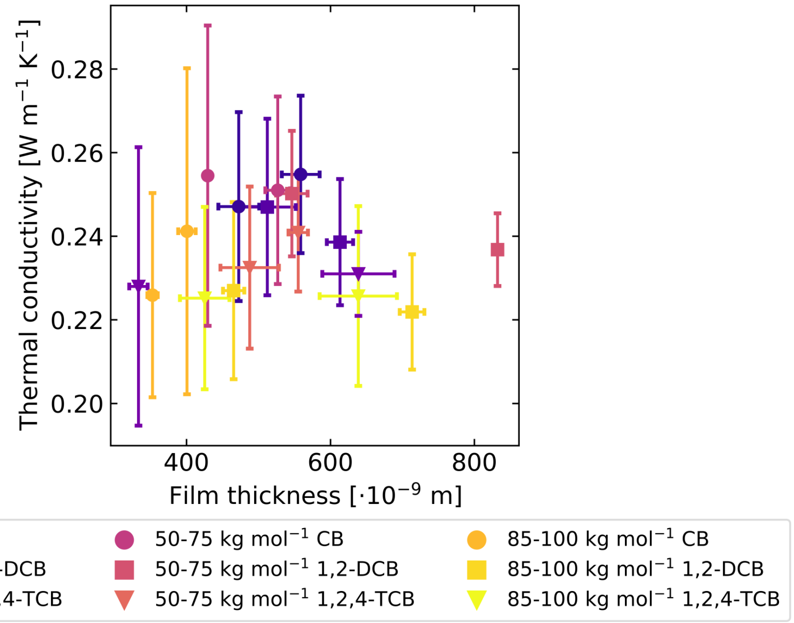

Figure S2. Thermal conductivity as a function of film thickness.

S3. Additional Correlations of Microstructure and Thermal Conductivity

Further correlations between microstructure parameters and thermal conductivity not shown in the main manuscript are shown in Figure S3. In this respect, there do not appear to be any direct correlations between the 0-0 transition energy or the Gaussian linewidth and the thermal conductivity in the range of measurement inaccuracies.

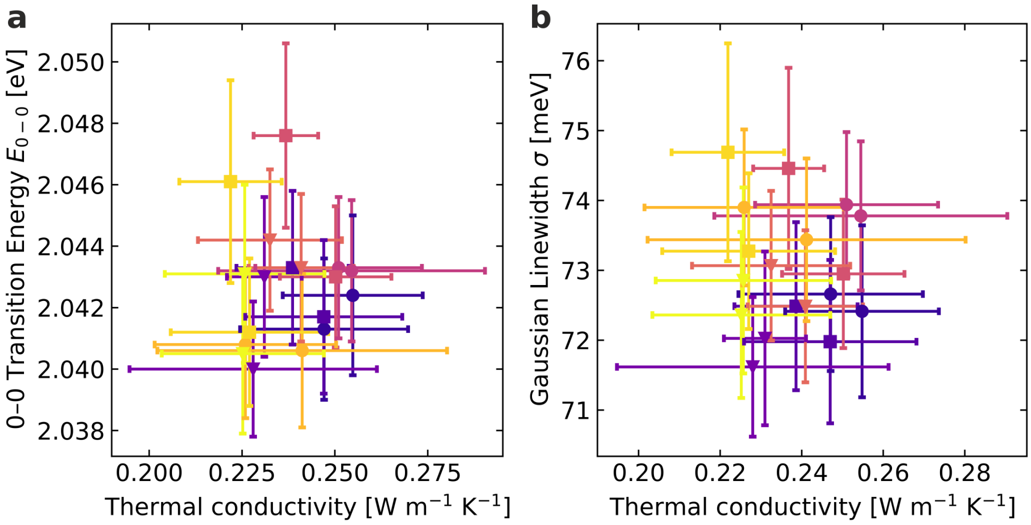

Figure S3. Correlations between extracted microstructural properties and thermal conductivity: **a** Relation between the 0-0 transition energy and the thermal conductivity. **b** Relation between the Gaussian linewidth and the thermal conductivity.

S4. GIWAXS of Quartz Reference

The background scattering of the GIWAXS measurements originating from substrate scattering is a broad feature as shown in the GIWAXS measurement of a Quartz substrate in Figure S4. This feature is found underneath the π-π stacking signal.

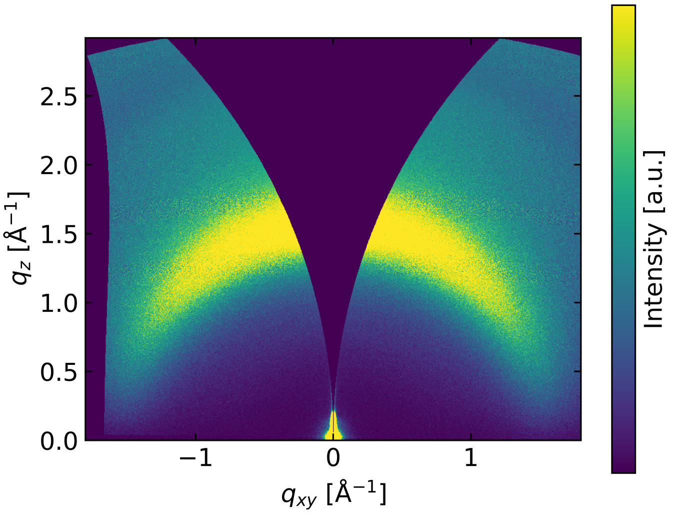

Figure S4. 2D GIWAXS data of the Quartz substrate.

S5. Additional Extracted GIWAXS Parameters

Figure S5 shows the peak widths of the π-π and the dominant lamellar peak from the GIWAXS measurements, two further fit parameters of the data presented in the main manuscript. The color code is analogous to that of the main manuscript.

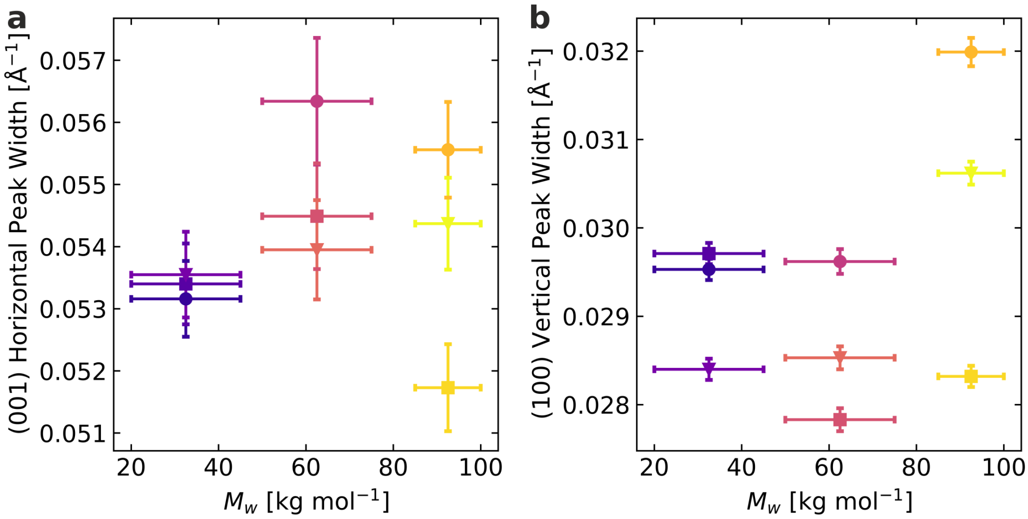

Figure S5. Fit parameters from GIWAXS data: **a** Peak width of the horizontal π-π stacking signal (001). **b** Peak width of the vertical lamellar stacking signal (100).
