# Supplementary figures and images for: Microstructural and Thermal Transport Properties of Regioregular Poly(3-hexylthiophene-2,5-diyl) Thin Films
Source: Materials (Basel). 2022 Nov 2;15(21):7700. doi: 10.3390/ma15217700 (PMC9654977; doi:10.3390/ma15217700)

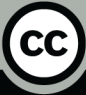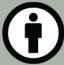

BY

Supplement: Supplementary file 1 [file materials-15-07700-s001.zip › Definitions/logo-ccby-eps-converted-to.pdf]

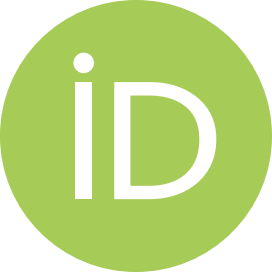

Supplement: Supplementary file 1 [file materials-15-07700-s001.zip › Definitions/logo-orcid-eps-converted-to.pdf]

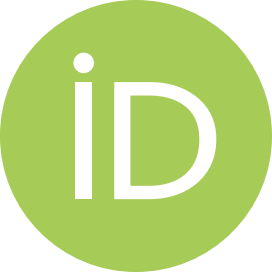

Supplement: Supplementary file 1 [file materials-15-07700-s001.zip › Definitions/logo-orcid.pdf]

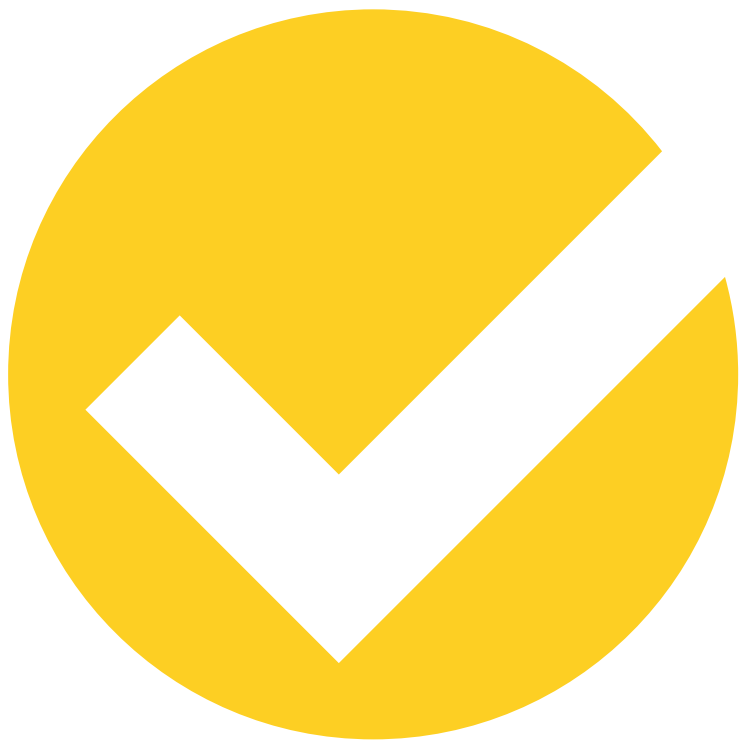

check for  
updates

Supplement: Supplementary file 1 [file materials-15-07700-s001.zip › Definitions/logo-updates.pdf]

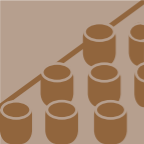

*materials*

Supplement: Supplementary file 1 [file materials-15-07700-s001.zip › Definitions/materials-logo-eps-converted-to.pdf]

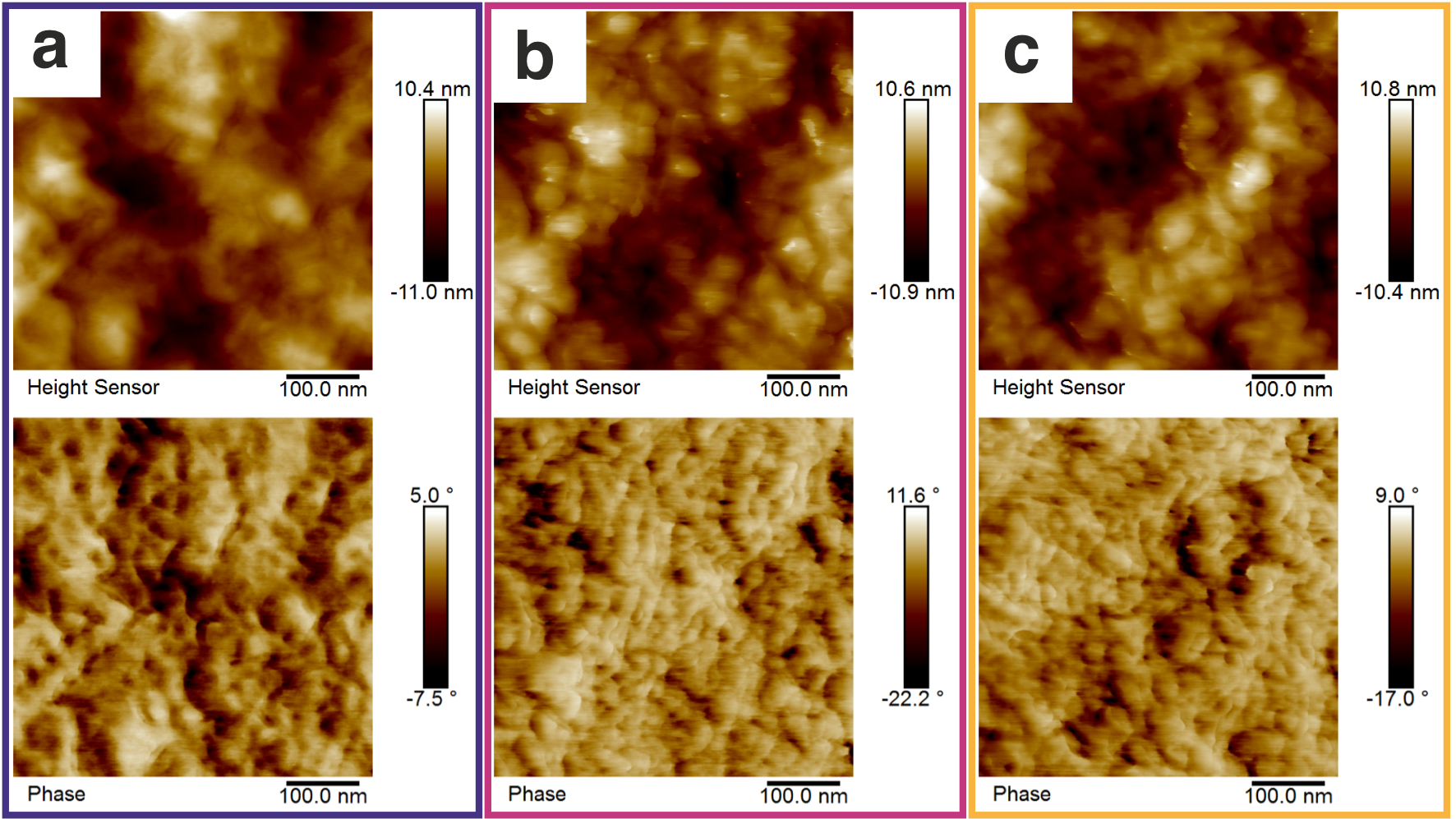

Supplement: Supplementary file 1 [file materials-15-07700-s001.zip › Definitions/SIAFM.png]

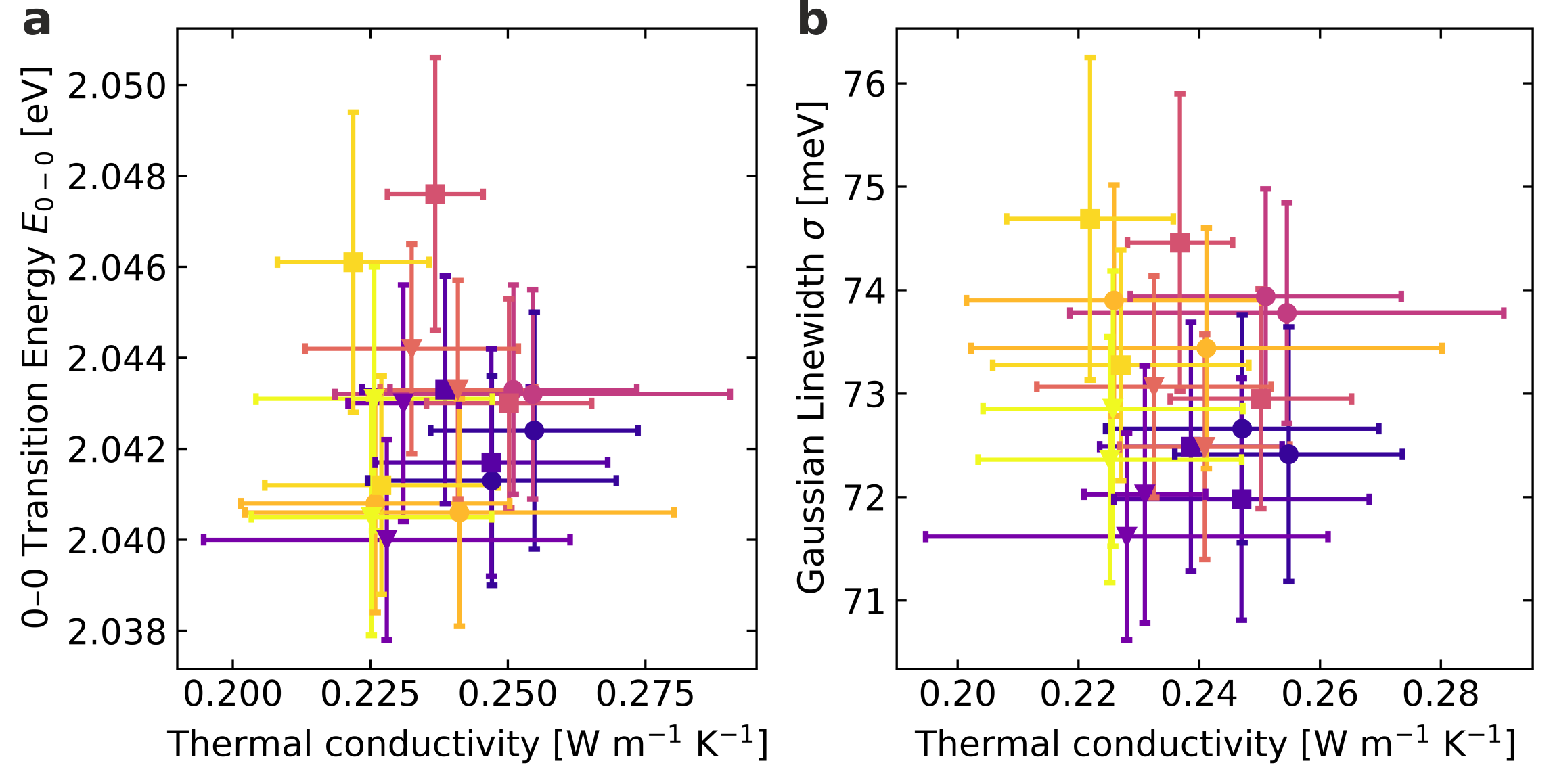

Supplement: Supplementary file 1 [file materials-15-07700-s001.zip › Definitions/SIcorrelations.png]

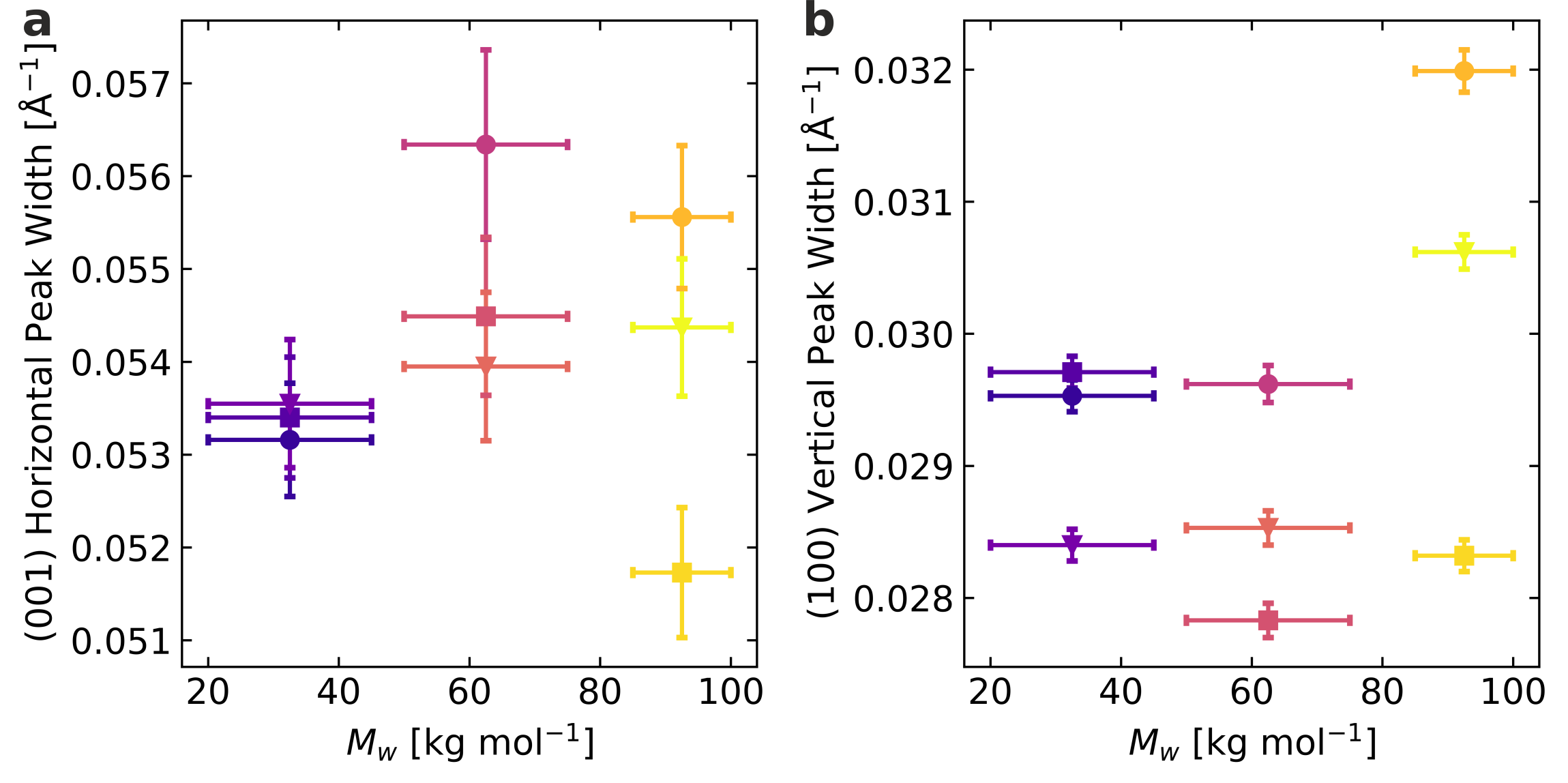

Supplement: Supplementary file 1 [file materials-15-07700-s001.zip › Definitions/SIGIWAXSFit.png]

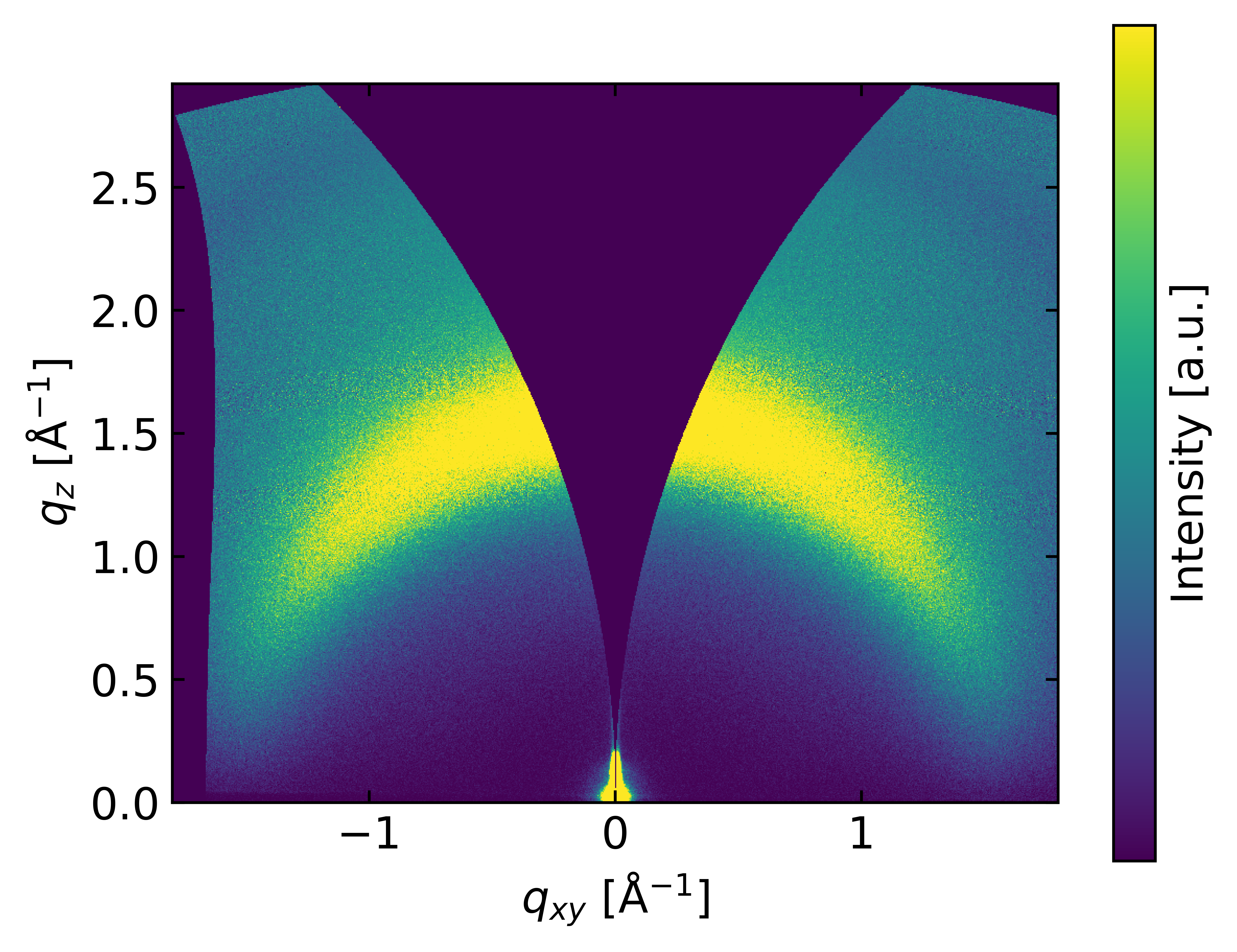

Supplement: Supplementary file 1 [file materials-15-07700-s001.zip › Definitions/SIGIWAXSQuartz.png]

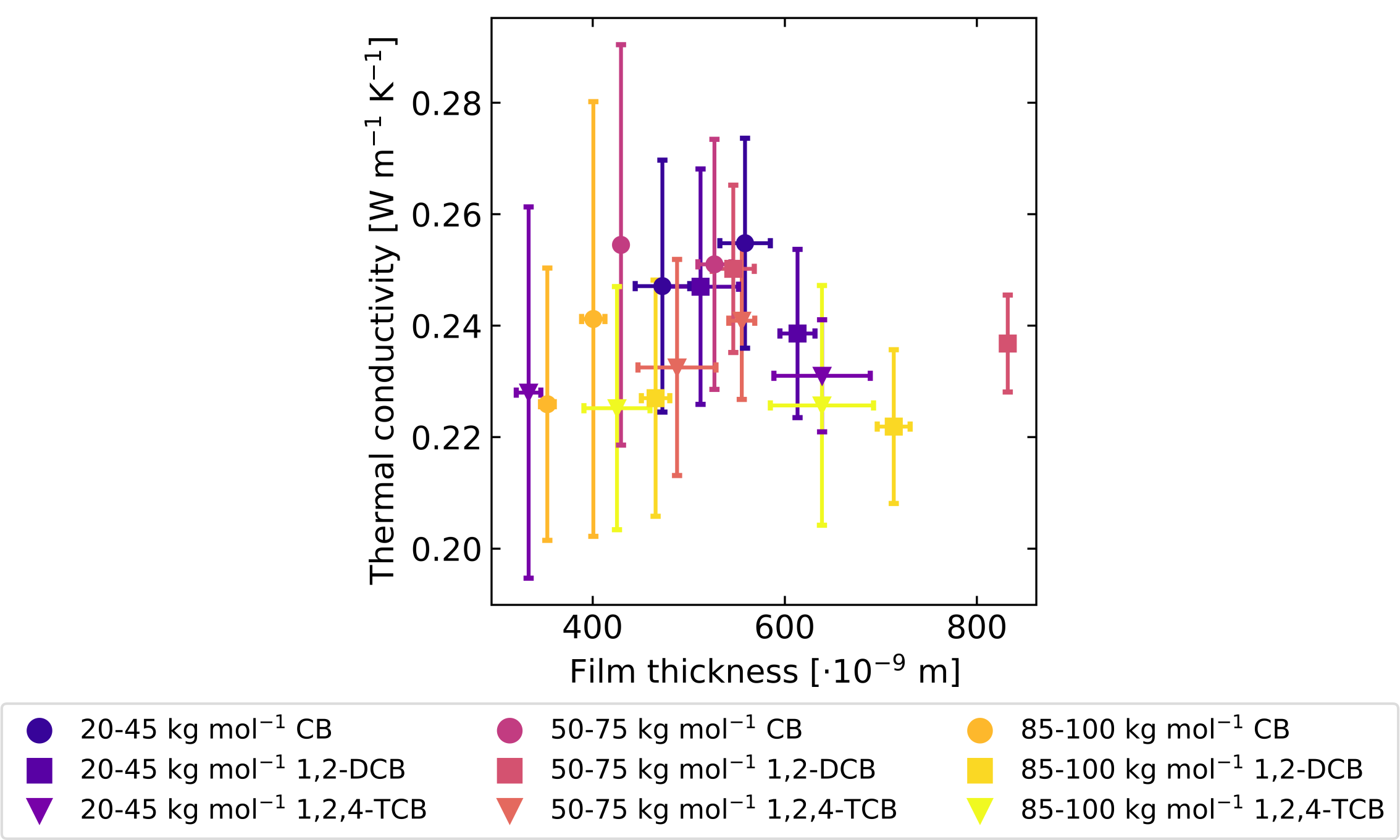

Supplement: Supplementary file 1 [file materials-15-07700-s001.zip › Definitions/SIthickness.png]
